# Facebook and Organ Transplantation

**Published:** 2012-08-01

**Authors:** G. S. Kalra

**Affiliations:** *Assistant Professor of Psychiatry, LTM Medical College and Sion Hospital, Mumbai, India*

**DEAR EDITOR,**

On May 1, 2012, the biggest social networking Web site, Facebook, started an initiative to connect various organ donors with people who need life-saving organ transplants [1]. Another encouraging start was when Mark Zuckerberg, the founder of Facebook, himself signed up first to be an organ donor. This case highlights two important issues in the field of organ transplantation. 

Firstly, this venture is a new attempt to use social networking Web sites as a new form of media in the field of organ transplantation. All other forms of media such as print, television, radio, *etc*. have been used and exhausted for making people aware of the importance of becoming organ donors. However, with the Internet becoming a significant extension of people’s lives today, it has still remained an untapped territory in organ transplantation until now. The social networking site, Facebook, connects people across the globe through various posts and wall messages. Facebook has more than 526 million daily users around the world [1]. It is obvious that with this huge membership, it is a definite fertile ground for initiating an action plan on something that should reach billions of people at the click of a mouse. 

The second important issue that this venture has touched upon is the interface of celebrity status and organ transplantation. With Facebook being the leading socializing network today, its founder Mark Zuckerberg has almost become a celebrity and is known widely across the world. It is a well-known fact that celebrities endorsing for organ donation is a good way to motivate people [2]. Therefore, the initiative that Mark took by himself signing up as the first organ donor can actually motivate many people to follow in his footsteps.

However, despite all the positive impacts that this venture is supposed to create around the world, one has to also consider the other side of the coin. Though the whole attempt looks quite helpful, one has to understand that there could be a lot of unnecessary clicks on these links by many who may not understand the depth of such a decision. On one hand, this may bring in people who have genuinely given a thought to the whole decision of becoming donors; on the other hand, there could be some people who just registered to belong to a gang under their peer pressure! This could later on falsely reflect as high number of registered donors with different implications that need to be considered. 

It has been claimed that this option was added to boost the number of potential organ donors; nevertheless, it will be worth a wait to see if this will be just another rat-race on the social networking site or will it bear some fruits in the long run.
